# Technoeconomic data and assumptions for long-term energy systems modelling in Indonesia

**DOI:** 10.1016/j.dib.2024.110323

**Published:** 2024-03-12

**Authors:** L.D. Hersaputri, R. Yeganyan, C. Cannone, F.A. Plazas-Niño, S. Osei-Owusu, Y. Kountouris, M. Howells

**Affiliations:** a Centre for Environmental Policy, Imperial College London, London, SW7 2BX United Kingdom; b STEER Centre, Department of Geography, Loughborough University, Loughborough, LE11 3TU United Kingdom; c Bartlett School of Environment, Energy and Resources, University College London, WC1E 6BT United Kingdom

**Keywords:** Renewable energy, Energy policy, Energy planning, Cost-optimisation, Decarbonisation

## Abstract

Indonesia's emission reduction commitment and clean energy transition target emphasises the importance of energy system modelling for analysing and projecting Indonesia's capacity, resource availability, and future conditions in achieving these objectives. Utilising energy systems modelling based on adequate and reliable data enables policymakers to select the most optimal alternatives in energy planning. Aligned with the U4RIA (Ubuntu, Retrievability, Repeatability, Reconstructability, Interoperability, Auditability) concept, this database may facilitate various related stakeholders in obtaining this comprehensive and detailed energy data, while the data gathering and processing can also be applied to other developing countries. This country-specific dataset covers the historical data of electricity generation, demand, installed capacity, capacity factor, technical lifetime, renewable energy potentials, costs, and its projections up to 2050. The data in this article is ready to be used for energy system and modelling research.

Specifications Table

**Table:** 

Subject	Energy
Specific subject area	Energy system modelling
Type of data	Tables and graphs.
Data collection	Data were collected from publicly accessible annual reports and databases from different energy related national institutions in Indonesia, as well as existing modelling databases. The annual reports and databases are available on related institutions’ website, listed on the Reference section.
Data source location	Raw data sources are listed in the different sections of this article, including previous dataset [8] and national institutions: • Ministry of Energy and Mineral Resources, Jakarta, Indonesia [8,9] • Indonesia's National Energy Council, Jakarta, Indonesia [11]
Data accessibility	With this article and in a repository Repository name: Zenodo Data identification number: 10.5281/zenodo.10369495 Direct URL to data: https://zenodo.org/records/10369495
Related research article	https://www.mdpi.com/2225-1154/12/3/37

## 1 Value of the Data

• This dataset can be used to develop energy system models and explore clean energy transition pathways in Indonesia. Incorporating this with various scenario framework and hypotheses may provide insights to policymakers.
• The data are open-source, comprehensive, and accessible, addressing challenges associated with complex and time-consuming data collection process.
• The data are useful for energy modellers, analysts, researchers, policymakers, and other related stakeholders as a foundation for model development and analysis in the energy sector. Moreover, the energy system analysis result enables governments to strategically allocate financial resources for implementation, define the role of public funds, and enhance accessibility to global climate finance [1].

## 2 Background

Energy systems modelling plays a crucial role in providing information and insights for policymakers. However, obtaining accurate and reliable data for national-scale modelling poses challenges due to inaccessibility and inconsistency, with an addition of costly energy modelling tools [2]. Thus, the present study addresses the aforementioned data gap, presenting energy data and assumptions for long-term energy planning in Indonesia that may be utilised by stakeholders from academia, public, and private sectors. In compliance with U4RIA concept of Ubuntu, Retrievability, Repeatability, Reconstructability, Interoperability, Auditability [3], this paper aims to improve energy modelling to support policy and decision-making in energy sector. This data-in-brief is the dataset used for research titled “Reducing Fossil Fuel Dependence and Exploring Just Energy Transition Pathways in Indonesia using OSeMOSYS (Open-Source Energy Modelling System)” [4] which focused on the data published by national institutions and existing model databases, as opposed to generic data from international organisations, to avoid inconsistency. This dataset may also be a starting point for future databases of other emerging nations, to be integrated to a new starter data-kit and building up the existing Starter Data Kits library [5] with a user-friendly ClicSAND (Simple and Nearly Done) interface [6].

## 3 Data Description

This article presents national datasets of Indonesia that can be utilised for energy modelling of a long-term decarbonisation and clean energy transition planning in OSeMOSYS tool. However, it is important to note that the data provided in this document exist independently of the tool. To enhance accessibility, the dataset can be accessed on Zenodo repository through the following link: https://zenodo.org/records/10369495 [7]. The data is sourced from publicly accessible sources, such as national institutions in Indonesia and pre-existing model databases. This contains information of costs (capital and fixed), capacity factor, technical life of power plants, electricity production and demand, installed capacity, and renewable energy supply and potential in 2015–2050, categorised into 9 technologies of existing power generators in Indonesia, defined in the excel file of repository under the sheet ‘Sets’.

### 3.1 Electricity demand and capacity factor

**Fig. 1:**
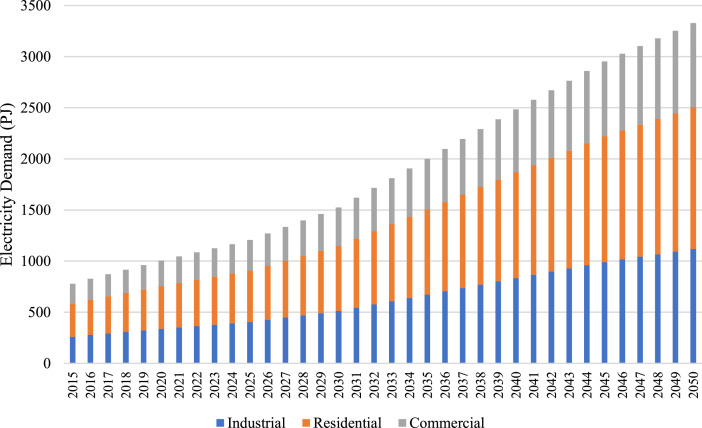
Electricity demand by sector (PJ).

Electricity demand data is sourced from pre-existing model database by Paiboonsin (2023) [8]. Electricity demand is classified through 3 (three) sectors, industrial, residential, and commercial, as shown on Table 1 and Fig. 1. The demand is forecasted to increase by 2–5 % annually. The data is available in the excel file of repository under the sheet ‘Demand’.

**Table 1:** Total Electricity Demand of Key Years (PJ).

Demand	2015	2020	2025	2030	2035	2040	2045	2050
Industrial	261.17	337.55	405.16	512.09	671.76	834.24	991.25	1117.80
Residential	323.97	418.71	502.58	635.22	833.28	1034.82	1229.59	1386.57
Commercial	192.77	249.15	299.06	377.98	495.84	615.76	731.66	825.06
Total Electricity Demand	777.91	1005.42	1206.80	1525.29	2000.87	2484.82	2952.49	3329.43

Capacity factor represents the overall utilisation ratio of a power generator, through the energy generated over a given timeframe compared to its full capacity. Capacity factor is estimated through a calculation and assumption listed on Section 3.1 from a pre-existing model dataset [8]. The data is available on Table 2 and in the excel file of repository under the sheet ‘Capacity Factor & Demand’.

**Table 2:** Capacity Factor of Power Generation Technologies.

Technology	Time Slices	Capacity Factor
Biomass Power Plant	S101–S104	0.7
Coal Power Plant	S101–S104	0.75
Geothermal Power Plant	S101–S104	0.7
Light Fuel Oil Power Plant	S101–S104	0.25
Oil Fired Gas Turbine (SCGT)	S101–S104	0.25
Gas Power Plant (CCGT)	S101–S104	0.55
Gas Power Plant (SCGT)	S101–S104	0.55
Solar PV (Utility)	S101	0
	S102	0.369931481
	S103	0
	S104	0.372241848
Concentrated Solar Power with Storage	S101	0.05
	S102	0.3
	S103	0.05
	S104	0.3
Large, Medium, and Small Hydropower Plant	S101	0.551496241
	S102	0.551496241
	S103	0.42004426
	S104	0.42004426
Onshore Wind	S101	0.034587963
	S102	0.030465741
	S103	0.030161232
	S104	0.019900362
Offshore Wind	S101–S104	0.2
Nuclear Power Plant	S101–S104	0.825
Onshore Wind	S101	0.034587963
	S102	0.030465741
	S103	0.030161232
	S104	0.019900362
Utility-scale PV with 2-hour storage	S101	0.061655247
	S102	0.369931481
	S103	0.062040308
	S104	0.372241848
Onshore Wind Power Plant with Storage	S101–S104	0.034948126
Light Fuel Oil Standalone Generator (1 kW)	S101–S104	0.4
Solar PV (Distributed with Storage)	S101	0.061655247
	S102	0.369931481
	S103	0.062040308
	S104	0.372241848
Off-grid Hydropower	S101	0.551496241
	S102	0.551496241
	S103	0.42004426
	S104	0.42004426

### 3.2 Capital and fixed costs

The capital and fixed cost data is mainly sourced from Ministry of Energy and Mineral Resources (MEMR) of Indonesia's report “Technology Data for the Indonesian Power Sector: Catalogue for Generation and Storage of Electricity” [9]. The data stated on MEMR's report include data of biomass, coal, geothermal, light fuel oil, CCGT, SCGT, solar PV (utility), all hydropower types, onshore and offshore wind of 2020, 2030, and 2050. Meanwhile data of other technologies is obtained from Paiboonsin (2023) [8]. The complete data is available in the excel file of repository under the sheet ‘Capital Cost’ and ‘Fixed Cost’.

### 3.3 Technical lifetime

The technical lifetime defines the typical length of power plant's operational years. The technical lifetime data is obtained from Ministry of Energy and Mineral Resources (MEMR)’s report [9] for biomass, coal, geothermal, light fuel oil, CCGT, SCGT, solar PV (utility), all hydropower types, onshore and offshore wind. Data of other technologies are obtained from previous model dataset [8]. The complete data is available in the excel file of repository under the sheet ‘Operational Life’. The data is listed on Table 5.

### 3.4 Residual capacity

Residual capacity is annual installed capacity of power generation technologies. The on-grid residual capacity data of 2015–2021 is sourced from MEMR's “Electricity Statistics of Indonesia” report [10]. Meanwhile the remaining years and some of the available off-grid technologies, also the electricity transmission and distribution capacity are obtained from existing model dataset [8]. The complete data is available in the excel file of repository under the sheet ‘Residual Capacity’.

### 3.5 Electricity production

**Fig. 2:**
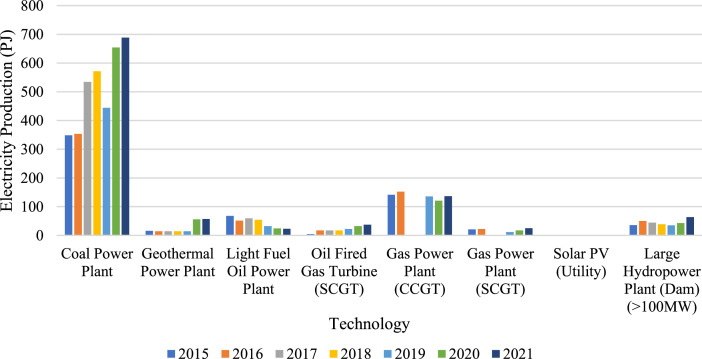
Historical electricity production by technology (PJ).

The historical electricity production data of each power generator in 2015–2021 is sourced from MEMR [10]. The data is also available in the excel file of repository under the sheet ‘Electricity Production’ (Fig. 2, Table 3, Table 4, Table 5, Table 6, Table 7, Table 8).

**Table 3:** Capital cost of key years ($/kW/year).

Technology	2015	2020	2025	2030	2035	2040	2045	2050
Biomass Power Plant	2000	2000	2000	1820	1820	1820	1820	1600
Coal Power Plant	1530	1530	1530	1480	1480	1480	1480	1430
Geothermal Power Plant	4500	4500	4500	3870	3870	3870	3870	3200
Light Fuel Oil Power Plant	800	800	800	800	800	800	800	780
Oil Fired Gas Turbine (SCGT)	1344	1344	1344	1344	1344	1344	1344	1344
Gas Power Plant (CCGT)	690	690	690	660	660	660	660	610
Gas Power Plant (SCGT)	770	770	770	730	730	730	730	680
Solar PV (Utility)	790	790	790	560	560	560	560	410
CSP with Storage	7404.71	4965.31	4000	3223.13	3178.59	3134.9	3134.9	3134.9
Large Hydropower Plant (Dam) (>100MW)	2080	2080	2080	2000	2000	2000	2000	1850
Medium Hydropower Plant (10–100MW)	2290	2290	2290	2200	2200	2200	2200	2040
Small Hydropower Plant (<10MW)	2700	2700	2700	2590	2590	2590	2590	2400
Onshore Wind	1500	1500	1500	1280	1280	1280	1280	1080
Offshore Wind	3500	3500	3500	2980	2980	2980	2980	2520
Nuclear Power Plant	5500	5500	5500	5500	5500	5500	5500	5500
Utility-scale PV with 2 hour storage	2785.5	1869	1287.33	1079.83	975.44	877.622	877.622	812.622
Onshore Wind power plant with storage	3294.52	2466.36	1935.13	1736.61	1496.17	1484.71	1484.71	1462.43
Light Fuel Oil Standalone Generator (1 kW)	1500	1500	1500	1500	1500	1500	1500	1500
Solar PV (Distributed with Storage)	3502	2130.8	1880.8	1755.8	1723.8	1690.8	1690.8	1625.8
Off-grid Hydropower	2162	2162	2162	2162	2162	2162	2162	2162
Electricity Transmission	204.267	204.267	204.267	204.267	204.267	204.267	204.267	204.267
Electricity Distribution	102.133	102.133	102.133	102.133	102.133	102.133	102.133	102.133

**Table 4:** Fixed cost of key years ($/kW/year).

Technology	2015	2020	2025	2030	2035	2040	2045	2050
Biomass Power Plant	47.6	47.6	47.6	43.8	43.8	43.8	43.8	38.1
Coal Power Plant	47.7	47.7	47.7	46.267	46.267	46.267	46.267	44.833
Geothermal Power Plant	57.5	57.5	57.5	49.45	49.45	49.45	49.45	40.85
Light Fuel Oil Power Plant	8	8	8	8	8	8	8	7.76
Oil Fired Gas Turbine (SCGT)	18	18	18	18	18	18	18	18
Gas Power Plant (CCGT)	23.5	23.5	23.5	22.8	22.8	22.8	22.8	22.1
Gas Power Plant (SCGT)	23.2	23.2	23.2	22.5	22.5	22.5	22.5	21.8
Solar PV (Utility)	14.4	14.4	14.4	10	10	10	10	8
CSP with Storage	120	120	120	120	120	120	120	120
Large Hydropower Plant (Dam) (>100MW)	37.7	37.7	37.7	36.2	36.2	36.2	36.2	33.6
Medium Hydropower Plant (10–100MW)	41.9	41.9	41.9	40.2	40.2	40.2	40.2	37.3
Small Hydropower Plant (<10MW)	53	53	53	50.9	50.9	50.9	50.9	47.2
Onshore Wind	60	60	60	51	51	51	51	43.2
Offshore Wind	72.6	72.6	72.6	61.7	61.7	61.7	61.7	52.3
Nuclear Power Plant	138	138	138	138	138	138	138	138
Utility-scale PV with 2 hour storage	27.855	18.69	12.8733	10.7983	9.7544	8.77622	8.44622	8.12622
Onshore Wind power plant with storage	131.781	98.6544	77.4052	69.4644	59.8468	59.3884	58.9137	58.497
Light Fuel Oil Standalone Generator (1 kW)	38	38	38	38	38	38	38	38
Solar PV (Distributed with Storage)	70.04	42.616	37.616	35.116	34.476	33.816	33.156	32.516
Off-grid Hydropower	64.86	64.86	64.86	64.86	64.86	64.86	64.86	64.86
Electricity Transmission	4.08533	4.08533	4.08533	4.08533	4.08533	4.08533	4.08533	4.08533
Electricity Distribution	2.04267	2.04267	2.04267	2.04267	2.04267	2.04267	2.04267	2.04267

**Table 5:** Technical lifetime (Years).

Technology	Technical Lifetime (Years)
Biomass Power Plant	25
Coal Power Plant	30
Geothermal Power Plant	30
Light Fuel Oil Power Plant	25
Oil Fired Gas Turbine (SCGT)	50
Gas Power Plant (CCGT)	25
Gas Power Plant (SCGT)	25
Solar PV (Utility)	35
CSP with Storage	35
Large Hydropower Plant (Dam) (>100MW)	50
Medium Hydropower Plant (10–100MW)	50
Small Hydropower Plant (<10MW)	50
Onshore Wind	27
Offshore Wind	27
Nuclear Power Plant	60
Utility-scale PV with 2 hour storage	30
Onshore Wind power plant with storage	30
Light Fuel Oil Standalone Generator (1 kW)	20
Solar PV (Distributed with Storage)	41
Off-grid Hydropower	40
Electricity Transmission	50
Electricity Distribution	70

**Table 6:** Residual capacity of key years (GW/year).

Technology	2015	2020	2025	2030	2035	2040	2045	2050
Biomass Power Plant	1.74	1.762	1.74	1.74	0	0	0	0
Coal Power Plant	27.23	36.668	34.3975	34.3975	34.3975	34.3975	34.3975	34.3975
Geothermal Power Plant	1.435	2.131	0	0	0	0	0	0
Light Fuel Oil Power Plant	6.275	4.864	4.987	4.987	4.987	4.987	4.987	4.987
Oil Fired Gas Turbine (SCGT)	0.819	3.178	3.207	3.207	3.207	3.207	3.207	3.207
Gas Power Plant (CCGT)	10.146	12.236	13.799	13.799	13.799	13.799	13.799	13.799
Gas Power Plant (SCGT)	4.311	5.348	5.348	5.348	5.348	5.348	5.348	5.348
Solar PV (Utility)	0.009	0.1473	0.2011	0.2011	0.2011	0.2011	0.2011	0.2011
Large Hydropower Plant (Dam) (>100MW)	5.079	5.638	4.53941	4.53941	4.53941	4.53941	4.53941	4.53941
Medium Hydropower Plant (10–100MW)	0.151	0.413	0.505	0.505	0.505	0.505	0.505	0.505
Small Hydropower Plant (<10MW)	0.03	0.106	0.042	0.042	0.042	0.042	0.042	0.042
Onshore Wind	0.00112	0.15431	0	0	0	0	0	0
Solar PV (Distributed with Storage)	0.04162	0.04508	0.04447	0.04287	0.02926	0.00337	0.00207	0.00207
Off-grid Hydropower	0.01442	0.01485	0.01485	0.01485	0.01485	0.01203	0.01025	0.00771
Electricity Transmission	58.9	58.9	58.9	58.9	58.9	58.9	58.9	58.9
Electricity Distribution	58.9	58.9	58.9	58.9	58.9	58.9	58.9	58.9

**Table 7:** Historical electricity production of power generator technologies (PJ).

Technology	2015	2016	2017	2018	2019	2020	2021
Coal Power Plant	348.32	353.74	534.11	571.59	444.15	653.89	688.08
Geothermal Power Plant	15.81	14.25	14.75	14.45	14.8	56.03	57.23
Light Fuel Oil Power Plant	67.89	51.61	59.52	54.63	32.59	24.23	23.12
Oil Fired Gas Turbine (SCGT)	4.44	17.23	17.23	17.23	22.15	32.69	37.27
Gas Power Plant (CCGT)	141.54	152.56	N/A	N/A	135.93	121.22	136.71
Gas Power Plant (SCGT)	21.27	22.31	N/A	N/A	11.57	17.11	25.15
Solar PV (Utility)	0.02	0.02	0.02	0.02	0.02	0.02	0.43
Large Hydropower Plant (Dam) (>100MW)	36.02	49.99	44.73	38.62	35.56	43.02	63.9

**Table 8:** Renewable energy potential and realisation (GW).

Renewable Energy Resources	Potential	Realisation
Geothermal	23.9	2.3
Bioenergy	56.9	2.3
Wind	154.9	0.2
Hydropower	95	6.6
Solar	3294	0.2

### 3.6 Renewable energy potential

Renewable energy resources in Indonesia includes, but not limited to, geothermal, bioenergy, wind, hydropower, and solar technology. The available renewable energy potential and realisation data is obtained from National Energy Council of Indonesia's “Indonesia Energy Outlook” report [11]. The data is also available in the excel file of repository under the sheet ‘RE Supply & Potential’.

## 4 Experimental Design, Materials and Methods

Data is collected through secondary data collection and literature review. From the pre-existing model dataset, this updates data from national institutions including Ministry of Energy and Mineral Resources (MEMR) and National Energy Council (NEC) of Indonesia. The raw data is then analysed and processed as the input for the energy modelling. Data sources and process methods are as follows.

### 4.1 Electricity demand and capacity factor

Electricity demand data is a raw data obtained from Paiboonsin [8], meanwhile capacity factor data is derived from a calculation of time slices reduction from 8 [8] to 4, representing existing season variations in Indonesia as an equatorial country (dry season–November to March and wet season–April to October) and daily load period (day–06 a.m.to 06 p.m. and night–06 p.m. and 06 a.m.). Thus, obtaining 4 time slices of S101 (Dry Day), S102 (Dry Night), S103 (Wet Day), and S104 (Wet Night). Time slices reduction calculation refers to Cannone et al. (2022) [12].

### 4.2 Capital and fixed costs

Capital and fixed costs data in this study refers to the raw data available on Indonesia's Ministry of Energy and Mineral Resources (MEMR)’s report “Technology Data for the Indonesian Power Sector: Catalogue for Generation and Storage of Electricity” [9], where data of 2020, 2030, and 2050 are available, while other years are assumed to be constant over the values of previously available years.

### 4.3 Technical lifetime, electricity production, renewable energy potential

Technical lifetime, electricity production, and renewable energy potential data are raw data from national institutions and further analysed for this study. The data of technical lifetime is sourced from MEMR's report “Technology Data for the Indonesian Power Sector: Catalogue for Generation and Storage of Electricity” [9]. Renewable energy potential data is sourced from National Energy Council of Indonesia's “Indonesia Energy Outlook” report [11]. Meanwhile historical electricity production data until 2021 is the raw data obtained from MEMR's “Electricity Statistics of Indonesia” report [10] in Gigawatt-Hour (GWh), and then processed to be consistent with the unit used in OSeMOSYS, Petajoule (PJ).

### 4.4 Residual capacity

Historical residual capacity data until 2021 is sourced from MEMR's report “Electricity Statistics of Indonesia” [10] for biomass, coal, geothermal, light fuel oil, oil (SCGT), gas CCGT, gas SCGT, solar PV (utility), onshore wind, and hydro. For data from 2022 for all technologies and solar PV (distributed with storage), off-grid hydropower, electricity transmission and distribution are obtained from pre-existing model database [8].

## Limitations

The limitation includes the limited data on the off-grid electricity production and power plant capacity, and thus this article focuses mainly on the available on-grid data with a few off-grids. Incorporating other off-grid technologies may provide a more comprehensive energy modelling analysis for long-term plan.

## Ethics Statement

The authors of this dataset have adhered to the ethical standards for publication in Data in Brief. They affirm that the study does not include participation of human subjects, animal experiments, or data from social media platforms.

## U4RIA Compliance Statement

This work follows the U4RIA guidelines which provide a set of high-level goals relating to conducting energy system analyses in countries [3]. This paper was carried out involving stakeholders in the development of models, assumptions, scenarios and results (Ubuntu / Community). The authors ensure that all data, source code and results can be easily found, accessed, downloaded, and viewed (retrievability), licensed for reuse (reusability), and that the modelling process can be repeated in an automatic way (repeatability). The authors provide complete metadata for reconstructing the modelling process (reconstructability), ensuring the transfer of data, assumptions and results to other projects, analyses, and models (interoperability), and facilitating peer-review through transparency (auditability).

## CRediT authorship contribution statement

**L.D. Hersaputri:** Conceptualization, Methodology, Formal analysis, Investigation, Data curation, Writing – original draft. **R. Yeganyan:** Project administration, Writing – review & editing. **C. Cannone:** Writing – review & editing. **F.A. Plazas-Niño:** Writing – review & editing. **S. Osei-Owusu:** Writing – review & editing. **Y. Kountouris:** Supervision. **M. Howells:** Supervision.

## Data Availability

Techno-Economic Dataset for Energy System Modelling in Indonesia (Original data) (Zenodo).
